# Divergent pathophysiological drivers of polycystic ovary syndrome: insulin resistance independently fuels the hyperandrogenic phenotype whilst neuroendocrine factors dominate non-hyperandrogenic presentations

**DOI:** 10.3389/fendo.2026.1758861

**Published:** 2026-02-04

**Authors:** Xiaoxia Wang, Hua Nie, Rong Cui, Guifang Ye, Ying Tan, Jing Zhang, Biyun Zhang, Xingming Zhong

**Affiliations:** 1 National Key Laboratory of Male Reproductive Genetics, Guangzhou,, China; 2 Reproductive Immunology, Guangdong Provincial Institute of Reproductive Sciences (Guangdong Provincial Fertility Hospital), Guangzhou,, China; 3 Central Laboratory, Guangdong Provincial Institute of Reproductive Sciences (Guangdong Provincial Fertility Hospital), Guangzhou,, China; 4 Centre for Reproductive Medicine, Guangdong Provincial Institute of Reproductive Sciences (Guangdong Provincial Fertility Hospital), Guangzhou,, China; 5 Department of Gynaecology, Guangdong Provincial Institute of Reproductive Sciences (Guangdong Provincial Fertility Hospital), Guangzhou, China

**Keywords:** body mass index, clinical phenotype, hyperandrogenism, insulin resistance, polycystic ovary syndrome (PCOS)

## Abstract

**Background:**

Polycystic Ovary Syndrome (PCOS) manifests as a heterogeneous disorder, yet the extent to which metabolic dysfunction drives specific phenotypes independent of obesity remains debated. This study aimed to delineate the distinct clinical and pathophysiological characteristics of Hyperandrogenic (HA) versus 38 Non-Hyperandrogenic (Non-HA) phenotypes, with a specific focus on disentangling the roles of insulin resistance and adiposity in driving androgen excess.

**Methods:**

A retrospective cross-sectional study was conducted involving 301 women with PCOS and 144 controls. Patients were stratified into Non-HA ( n=49) and HA ( n=252) subgroups based strictly on comprehensive androgen profiling (biochemical and clinical assessment). We utilised multivariate logistic regression to identify independent predictors of the HA phenotype and stratified linear regression models to map the relationships between metabolic indices (HOMA-IR, BMI) and reproductive parameters.

**Results:**

The HA phenotype was characterised by significantly more severe oligo-anovulation and metabolic disturbance compared to the Non-HA group, despite comparable age ( P=0.069). Multivariate analysis adjusted for potential confounders revealed that HOMA-IR was a robust, independent predictor of the HA phenotype (aOR=1.35, P=0.003), comparable to LH (aOR=1.09, P=0.012). Crucially, Body Mass Index (BMI) failed to retain statistical significance (aOR=0.98, P=0.682) in the adjusted model, indicating that the association between metabolic dysfunction and hyperandrogenism is not mediated solely by adiposity. Stratified linear regression further demonstrated a distinct positive trajectory between HOMA-IR and testosterone specifically within the HA cohort ( $R^{2}=0.20,P=0.013$), a relationship absent in the Non-HA group. Conversely, in Non-HA patients, menstrual cycle prolongation correlated with LH levels rather than metabolic markers, suggesting a predominant neuroendocrine aetiology.

**Conclusion:**

Our findings demonstrate that PCOS encompasses two pathophysiologically distinct entities. The Non-HA phenotype appears driven primarily by neuroendocrine dysregulation, whereas the HA phenotype is intrinsically linked to metabolic dysfunction, specifically insulin resistance. Most importantly, we confirm that insulin resistance drives the hyperandrogenic phenotype independently of obesity. These data support a paradigm shift towards phenotype-specific management, necessitating aggressive insulin-sensitising strategies for hyperandrogenic patients regardless of their BMI.

## Introduction

Polycystic Ovary Syndrome (PCOS) stands as the pre-eminent endocrine-metabolic disorder affecting women of reproductive age, with a global prevalence estimated at 8–13% (1, 2). Governed by the Rotterdam criteria (3), the diagnosis encompasses a broad spectrum of clinical presentations, requiring at least two of three cardinal features: oligo-anovulation, clinical and/or biochemical hyperandrogenism, and polycystic ovarian morphology (4–6). This diagnostic heterogeneity suggests that PCOS is not a monolithic disease entity but rather a complex syndrome with divergent phenotypic expressions, ranging from mild reproductive dysfunction to severe metabolic perturbation. Within this spectrum, Hyperandrogenism (HA) is widely regarded as the central pathogenic driver, underpinning the syndrome’s reproductive and metabolic sequelae (1, 7). Preclinical models corroborate this centrality, demonstrating that excess androgen exposure recapitulates key PCOS traits, including neuroendocrine defects and insulin resistance (8, 9).

However, the phenotypic architecture of PCOS remains a subject of intense debate. A significant proportion of women diagnosed with PCOS—approximately 20% to 30%—present with normal circulating androgen levels and absence of clinical signs, suggesting that alternative pathophysiological trajectories, potentially distinct from primary androgen excess, underpin the clinical presentation (10–12). This variability points towards divergent metabolic and neuroendocrine set-points governing the different phenotypes (1, 4, 8). Current evidence increasingly elucidates the “vicious cycle” linking hyperinsulinaemia and hyperandrogenism: insulin resistance not only correlates with adiposity but also exerts a co-gonadotrophic effect, synergising with Luteinising Hormone (LH) to amplify thecal androgen production (13–15). Crucially, this pathogenic impact is systemic; hyperinsulinaemia concurrently heightens the adrenal steroidogenic response to ACTH stimuli and suppresses hepatic synthesis of Sex Hormone Binding Globulin (SHBG), culminating in a net increase in both total and free androgen bioavailability (16, 17). This metabolic-reproductive coupling creates a stratified metabolic hierarchy across the syndrome. Indeed, insulin resistance has been identified as a phenotype-specific trait that follows a graded scale: it is an intrinsic feature of hyperandrogenic presentations—most profound in the classic phenotype and evident in ovulatory hyperandrogenism—yet is notably attenuated in the Non-HA phenotype (18). Consequently, women harbouring these androgen-excess profiles carry a disproportionately elevated risk of developing type 2 diabetes and cardiovascular disease compared to their normoandrogenic counterparts (19, 20).

Despite these advances, critical gaps persist in our understanding of how specific hormonal and metabolic drivers segregate across phenotypes. The extent to which Insulin Resistance (IR) acts as an independent driver of hyperandrogenism versus a mere bystander of obesity remains contentious (21–24). Furthermore, it is unclear whether the neuroendocrine dysregulation (e.g., elevated LH) and ovarian morphological burden differ fundamentally between HA and Non-HA subgroups, or if they represent a continuum of severity (23, 24). Resolving these uncertainties is essential for shifting from a “one-size-fits-all” management approach toward precision medicine.

To address these complexities, we conducted a comprehensive retrospective cross-sectional study to delineate the distinct pathophysiological signatures associated with the hyperandrogenic phenotype. Unlike previous studies that often conflate heterogeneous phenotypes, we employed a stratified parallel comparison strategy. We contrasted the clinical, hormonal, and metabolic profiles of HA and Non-HA patients against a healthy control cohort, with a specific focus on dissecting the independent roles of HOMA-IR and LH dynamics. By adopting this phenotype-centred lens, we aim to elucidate the heterogeneous mechanisms driving PCOS, clarify the contribution of metabolic dysfunction to androgen excess, and provide evidence to support more nuanced, pathophysiology-based risk stratification in clinical practice.

## Materials and methods

### Study design and participants

We conducted a retrospective cross-sectional study involving 445 women recruited from the Clinic of Reproductive Immunology at Guangdong Provincial Reproductive Hospital between January 2013 and November 2015. The study population comprised 301 women with a confirmed diagnosis of Polycystic Ovary Syndrome (PCOS) and a control group of 144 age-matched healthy women.

The diagnosis of PCOS was established in accordance with the revised Rotterdam consensus criteria (3), requiring the presence of at least two of the following three features: oligo-ovulation or anovulation, clinical and/or biochemical signs of hyperandrogenism, and polycystic ovarian morphology (PCOM) on ultrasound. Specifically, PCOM was defined by a follicle number per ovary (FNPO) of ≥ 12 and/or an ovarian volume of ≥ 10 mL in at least one ovary, assessed via transvaginal ultrasonography in the absence of corpora lutea, cysts, or dominant follicles. Consistent with international guidelines, we rigorously excluded subjects with other aetiologies of androgen excess or ovulatory dysfunction. Specific exclusion criteria encompassed non-classic congenital adrenal hyperplasia (screened via 17-OHP), thyroid dysfunction, hyperprolactinaemia, Cushing’s syndrome, and hypogonadotropic hypogonadism, specifically functional hypothalamic amenorrhoea (FHA). FHA was meticulously ruled out based on a comprehensive clinical assessment of energy balance (e.g., absence of recent weight loss, excessive exercise, or chronic stress) and hormonal profiling (exclusion of profoundly low gonadotrophins and oestradiol in the presence of amenorrhoea).

The control group consisted of women seeking reproductive advice solely for male-factor infertility or tubal factors. Inclusion criteria for controls were the presence of regular menstrual cycles ranging from 21 to 35 days, the absence of clinical or biochemical evidence of hyperandrogenism, and ultrasonographically normal ovaries. Women with a history of hypertension, cardiovascular disease, diabetes mellitus, or those taking hormonal medications (including oral contraceptives or insulin sensitisers) within three months prior to recruitment were excluded from both groups.

### Ethical statement

The study protocol was reviewed and approved by the Ethics Committee of Guangdong Provincial Reproductive Hospital. Given the retrospective nature of the analysis using de-identified medical records, the requirement for written informed consent was waived by the institutional review board. The study was conducted in strict adherence to the Declaration of Helsinki regarding medical research involving human subjects and data confidentiality.

### Clinical and anthropometric measurements

Baseline demographic and clinical data were extracted from electronic medical records. Anthropometric measurements, including height and weight, were obtained by trained nursing staff using calibrated instruments. Body Mass Index (BMI) was calculated as weight in kilograms divided by the square of height in metres (weight (kg)/height² (m²)). Hirsutism was assessed clinically using the modified Ferriman-Gallwey score. Phenotype stratification for the purpose of this study incorporated both clinical signs and biochemical markers.

### Ultrasound assessment and morphological classification

Transvaginal ultrasound was performed to assess ovarian morphology. Strictly adhering to the 2003 Rotterdam criteria, Polycystic Ovarian Morphology (PCOM) was further categorized into three distinct morphological patterns based on ovarian volume (OV) and follicle number per ovary (FNPO). These were defined as follows: the Volume-dominant phenotype (coded as 0), characterized by an OV ≥ 10 mL with an FNPO < 12; the Follicle-dominant phenotype (coded as 1), characterized by an FNPO ≥ 12 with an OV < 10 mL; and the Mixed phenotype (coded as 2), characterized by meeting both criteria (OV ≥ 10 mL and FNPO ≥ 12). This classification ensured that all participants met the PCOM criteria through volume enlargement, follicle count, or both.

### Biochemical and hormonal assays

Venous blood samples were collected from the antecubital vein between 08:00 and 10:00 AM following an overnight fast of at least 8 hours. For all participants, sampling was performed during the early follicular phase, specifically on days 2 to 5 of a spontaneous or progesterone-induced menstrual cycle. Serum concentrations of Follicle-Stimulating Hormone (FSH), Luteinising Hormone (LH), Prolactin (PRL), Oestradiol (E2), and Total Testosterone (T) were quantified using a fully automated electrochemiluminescence immunoassay system (Roche Cobas e601/8866; Roche Diagnostics, Mannheim, Germany). Intra- and inter-assay coefficients of variation (CV) for all hormonal assays were consistently below 5% and 10%, respectively. Metabolic parameters, including Fasting Plasma Glucose (FPG), were measured using the hexokinase method. Fasting Insulin (FINS) was determined via electrochemiluminescence.

### Definition of subgroups and metabolic indices

Stratification of PCOS phenotypes was conducted using two distinct criteria. A composite Hyperandrogenic (HA) phenotype was defined by the presence of biochemical hyperandrogenism (elevated serum total testosterone level >1.97 nmol/L, referencing the 95th percentile of the specific population (26)) and/or clinical signs (e.g., hirsutism). Patients lacking both biochemical and clinical evidence of hyperandrogenism, but fulfilling the other Rotterdam criteria (i.e., Oligo-anovulation + PCOM), were classified as the Non-Hyperandrogenic (Non-HA) phenotype. Insulin resistance was evaluated using the Homeostasis Model Assessment for Insulin Resistance (HOMA-IR), calculated using the formula: HOMA-IR = [fasting insulin (μIU/mL) × fasting glucose (mmol/L)]/22.5. A cut-off value of HOMA-IR > 2.69 was utilised to define insulin resistance, in accordance with established criteria for the adult population in this region.

### Statistical analysis

Data analysis was performed using SPSS software (Version 26.0; IBM Corp., Armonk, NY, USA) and GraphPad Prism (Version 9.0; GraphPad Software, San Diego, CA, USA). The normality of continuous variables was assessed using the Shapiro-Wilk test and visual inspection of histograms. Descriptive statistics for normally distributed data are presented as mean ± standard deviation (SD), while non-normally distributed data are expressed as median with interquartile range (IQR). Categorical variables are reported as frequencies and percentages. Differences in continuous variables between groups (Control vs. PCOS; Non-HA vs. HA) were analysed using the independent Student’s t-test for parametric data or the Mann-Whitney U test for non-parametric data. Categorical variables were compared using the Chi-square test or Fisher’s exact test as appropriate.

To explore the interrelationships between metabolic and reproductive parameters, Spearman’s rank correlation matrices were generated for each phenotype. To identify independent predictors of the hyperandrogenic phenotype, univariate and multivariate logistic regression analyses were performed. The multivariate model was adjusted for potential confounders, including Age and BMI, and results are presented as Odds Ratios (OR) with 95% Confidence Intervals (CI). Additionally, linear regression models were fitted to visualise the relationship between HOMA-IR or BMI and Testosterone levels. To control for Type I errors in multiple comparisons, the Benjamini-Hochberg False Discovery Rate (FDR) correction was applied where indicated. A two-tailed *P*-value of less than 0.05 was considered statistically significant.

## Results

### Comparative characteristics between control and PCOS groups

The baseline demographic, clinical, and biochemical characteristics of the study population are summarised in **Table 1**. The PCOS cohort (n=301) and the control group (n=144) were comparable in terms of age (*P* = 0.070). However, regarding anthropometric parameters, the PCOS group exhibited a statistically higher body weight [Median (IQR): 60.5 (58.0, 67.0) kg vs. 55.0 (52.0, 62.0) kg; *P* < 0.001] and BMI [23.3 (22.0, 24.32) kg/m² vs. 22.4 (20.15, 24.8) kg/m²; *P* = 0.004] compared to the control group. Clinically, the PCOS patients demonstrated marked reproductive dysfunction, characterised by significantly prolonged menstrual cycles (62.8 ± 20.5 days) compared to the regular cycles observed in the control group (30.1 ± 2.2 days; *P* < 0.001).

**Table 1:** Comparative analysis of baseline clinical and biochemical characteristics between Control and PCOS groups.

Variables	Control group (n=144)	PCOS group (n=301)	*P*
Demographics & Anthropometrics			
Age	30.3 ± 4.4	28.9 ± 4.0	0.070
Weight (kg)	55.0 (52.0, 62.0)	60.5 (58.0, 67.0)	**<0.001**
BMI(kg/m^2^)	22.4 (20.15, 24.8)	23.3 (22.0, 24.32)	**0.004**
Menstrual cycles (days)	30.1 ± 2.2	62.8 ± 20.5	**<0.001**
Hormonal Profile			
FSH(mIU/mL)	5.20 ± 1.40	5.18 ± 1.29	0.888
LH(mIU/mL)	4.56 ± 1.81	7.99 ± 4.62	**<0.001**
LH/FSH ratio	0.92 ± 0.39	1.63 ± 1.40	0.364
E2(pmol/L)	134.29 ± 53.85	147.34 ± 179.13	0.114
PRL(mIU/L)	368.81 ± 131.53	437.03 ± 296.47	**<0.001**
T(nmol/L)	1.52 ± 0.88	2.71 ± 1.35	**<0.001**
Metabolic Profile			
HOMA_IR	1.65 ± 0.46	3.75 ± 2.16	0.070

Bold values denote statistical significance (*P* < 0.05).

Hormonal profiling revealed distinct neuroendocrine and androgenic aberrations in the PCOS cohort. LH levels were nearly twofold higher in patients with PCOS compared to controls (7.99 ± 4.62 mIU/mL vs. 4.56 ± 1.81 mIU/mL; *P* < 0.001). Similarly, T concentrations were significantly elevated in the PCOS group (2.71 ± 1.35 nmol/L vs. 1.52 ± 0.88 nmol/L; *P* < 0.001), confirming the hyperandrogenic status of the study subjects. Regarding prolactin, levels were significantly higher in the PCOS group (437.03 ± 296.47 mIU/L) compared to controls (368.81 ± 131.53 mIU/L; *P* < 0.001). However, it is noteworthy that despite this statistical elevation, the mean concentration in the PCOS group remained within our laboratory’s physiological reference range (102–496 mIU/L). Thus, this likely reflects a subtle neuroendocrine fluctuation inherent to the syndrome rather than pathological hyperprolactinaemia. Conversely, no statistically significant differences were observed in FSH, the LH/FSH ratio, or oestradiol levels between the two groups.

Regarding metabolic parameters, although the Homeostasis Model Assessment for HOMA-IR index appeared numerically higher in the PCOS cohort (3.75 ± 2.16) relative to controls (1.65 ± 0.46), this difference did not reach statistical significance in the overall cohort comparison (*P* = 0.070).

### Clinical and biochemical characteristics stratified by hyperandrogenic phenotype

**Figure 1:**
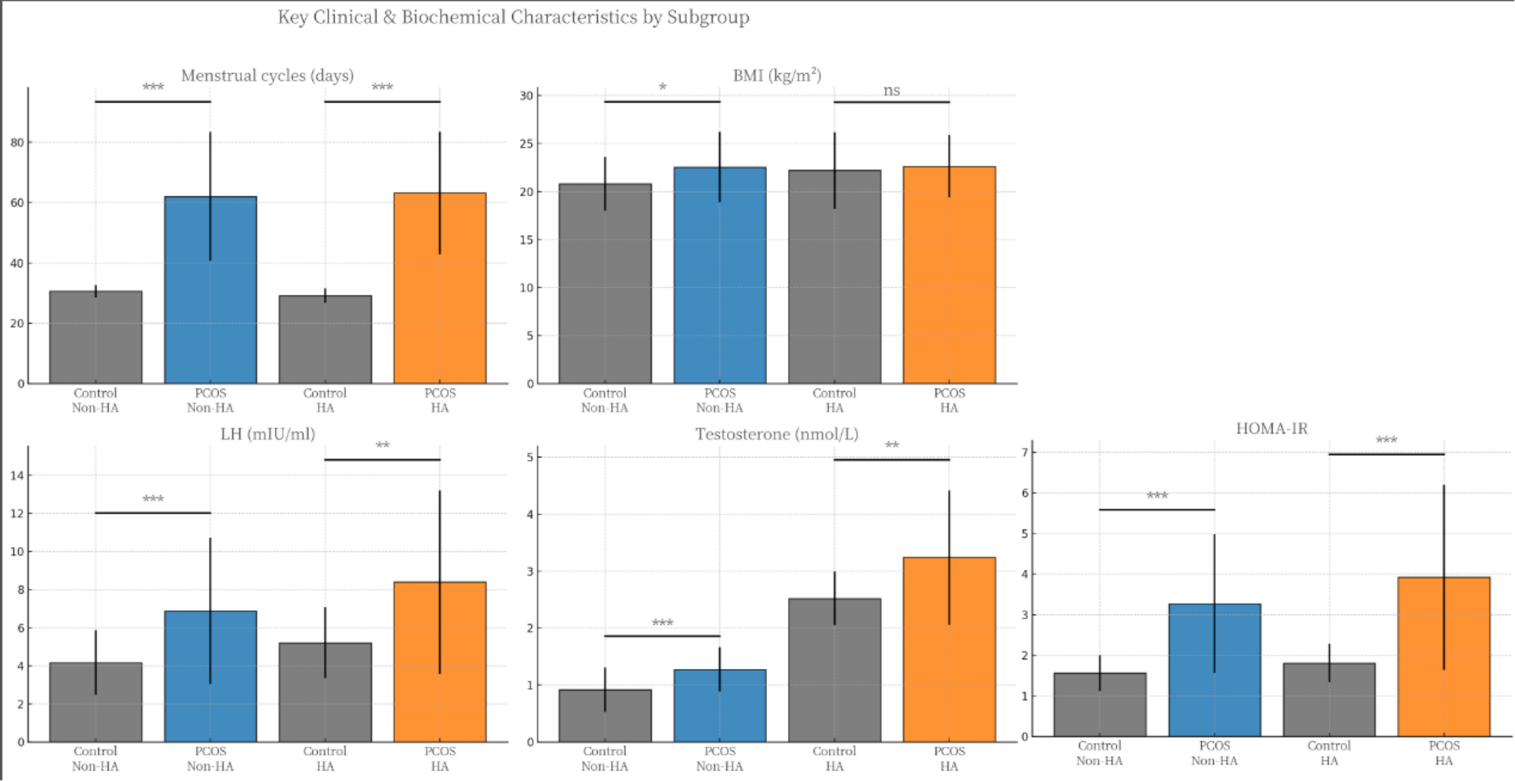
Comparative profiling of key clinical, hormonal, and metabolic parameters stratified by hyperandrogenic phenotype. Bar charts illustrate the differences in Menstrual Cycle Length, BMI, LH, T, and HOMA-IR levels between Control and PCOS subjects within Non-HA and HA subgroups. Data are presented as means, with error bars representing standard deviations (SD). Statistical comparisons were performed between the Control and PCOS groups within each specific phenotype. Significance levels: * *P* < 0.05, ** *P* < 0.01, *** *P* < 0.001; ns, not significant (*P* > 0.05).

To dissect the heterogeneity of PCOS, we stratified the study population into Non-HA and HA subgroups. The comparative analysis of these phenotypes against their respective controls is detailed in **Supplementary Table 1**, with key differentiators visualised in **Figure 1**. It is pertinent to acknowledge the disparity in sample sizes within the control arm, specifically the limited number of subjects in the HA control group (n=13) compared to the Non-HA control group (n=131). While this reflects the rarity of isolated hyperandrogenism in eumenorrhoeic women, statistical interpretations regarding this specific sub-cohort have been approached with appropriate prudence to ensure the validity of the conclusions.

Demographically, age distribution remained homogenous across all subgroups (Non-HA, P = 0.123; HA, *P* = 0.492). However, menstrual dysfunction was a universal feature of PCOS irrespective of the androgenic phenotype. As shown in **Figure 1**, menstrual cycle length was markedly prolonged in both Non-HA and HA cohorts compared to controls (*P* < 0.001 for both). Regarding body composition, a divergent pattern was observed: while Non-HA patients exhibited a significantly higher BMI compared to their controls (*P* = 0.038), no statistically significant difference in BMI was detected between the HA-PCOS group and the control group (*P* = 0.637; **Figure 1**). Neuroendocrine disturbances, characterised by elevated LH, were evident across the PCOS spectrum. Both Non-HA and HA subgroups demonstrated significantly higher LH levels compared to controls (*P* < 0.001). Consequently, the LH/FSH ratio was significantly elevated in both PCOS phenotypes (Non-HA, *P* = 0.035; HA, *P* = 0.004). FSH levels remained comparable across all groups. Predictably, serum testosterone concentrations were substantially augmented in the PCOS cohorts (**Figure 1**). This elevation was most pronounced in the HA subgroup (3.24 ± 1.18 nmol/L; *P* < 0.001 vs controls), confirming the severity of the androgenic phenotype, whereas the Non-HA subgroup showed a modest yet significant increase (1.27 ± 0.39 nmol/L; *P* < 0.001 vs controls).

Crucially, metabolic dysfunction appeared to transcend the androgenic classification. HOMA-IR was significantly elevated in both PCOS subgroups relative to their respective controls (*P* < 0.001 for both; **Figure 1**). Notably, despite the lack of significant BMI difference in the HA comparison, the HA group exhibited the highest degree of HOMA-IR (3.92 ± 2.28) compared to the Non-HA group (3.27 ± 1.71), suggesting that the HA phenotype may be associated with a more severe metabolic exacerbation independent of adiposity alone.

### Comparison of metabolic and reproductive characteristics between HA-PCOS and NA-PCOS phenotypes

**Figure 2:**
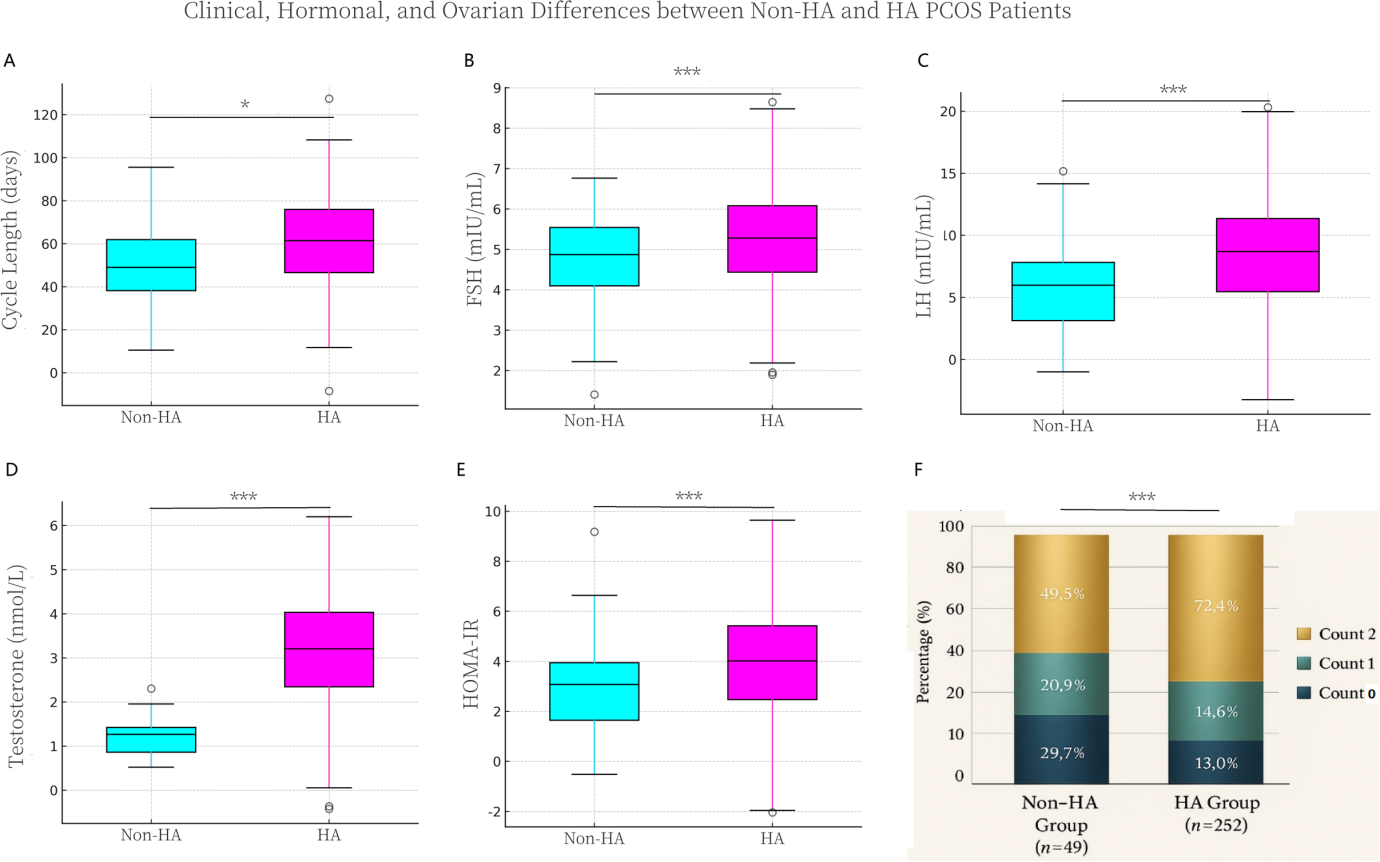
Distinctive clinical, hormonal, and ovarian morphological profiles of 760 Hyperandrogenic (HA) versus Non-Hyperandrogenic (Non-HA). **(A–E)** Box-and-whisker plots comparing key physiological parameters between Non-HA (Cyan) and HA (Magenta) subgroups. The central horizontal line represents the median, the box spans the interquartile range (IQR, 25th–75th percentiles), and the whiskers extend to the minimum and maximum values excluding outliers. Significant elevations were observed in the HA cohort for **(A)** Menstrual Cycle Length, **(B)** Follicle-Stimulating Hormone (FSH), **(C)** Luteinising Hormone (LH), **(D)** Total Testosterone, and **(E)** HOMA-IR. **(F)** Stacked bar chart illustrating the distribution of Polycystic Ovarian Morphology (PCOM) patterns. Scores are classified according to the specific morphological dominance: 0 = Volume-dominant phenotype (Ovarian Volume ≥ 10 mL with FNPO < 12); 1 = Follicle-dominant phenotype (FNPO ≥ 12 with Ovarian Volume < 10 mL); and 2 = Mixed phenotype (meeting both criteria). The HA phenotype was significantly associated with the Mixed morphology (Score 2), whereas the Non-HA group exhibited a higher proportion of the Volume-dominant phenotype (Score 0) (P < 0.001, Chi-square test). Significance levels: * *P* < 0.05, ** *P* < 0.01, *** *P* < 0.001.

To elucidate the specific impact of biochemical androgen excess on the PCOS spectrum, we stratified patients strictly based on androgen status. Although initial stratification relied on biochemical hyperandrogenism (HA group, **Supplementary Table 1**), the comprehensive phenotype assessment necessitated the reclassification of 32 patients—who presented with normal serum androgen levels yet distinct clinical signs (e.g., hirsutism)—into the HA cohort. Consequently, the definitive phenotypic analysis (**Supplementary Table 2**, **Figure 2**) comprised 49 patients in the Non-HA group and 252 patients in the HA group.

To elucidate the specific impact of androgen excess on the PCOS spectrum, we compared their clinical features and biochemical markers between these stratified cohorts. Comprehensive statistical comparisons are detailed in **Supplementary Table 2**, with key distinctive features visualised in **Figure 2**. Notably, while age was comparable between the two subgroups (*P* = 0.069), the HA phenotype was associated with a more severe disruption of reproductive function. As illustrated in **Figure 2**, menstrual cycle length was significantly prolonged in HA patients (61.2 ± 21.5 days) compared to the Non-HA group (54.2 ± 22.3 days; *P* = 0.008), indicative of profound oligo-anovulation.

This clinical observation was mirrored by distinct alterations in ovarian morphology (**Figure 2F**). Chi-square analysis revealed a significant difference in polycystic ovary patterns between the groups (P < 0.001). The HA cohort exhibited a “classic” mixed morphology, with 72.4% of patients presenting with both enlarged volume and increased follicle count (Score 2). In contrast, the Non-HA group had a significantly higher proportion of patients with the Volume-dominant phenotype (Score 0; 29.7% vs. 13.0%). It is crucial to clarify that under the 2003 Rotterdam criteria used in this study (3), Score 0 represents valid Polycystic Ovarian Morphology (PCOM) defined by ovarian enlargement (OV ≥ 10 mL) despite a follicle count < 12. Thus, for Non-HA patients presenting with this Volume-dominant phenotype, the diagnosis of PCOS was firmly established by the co-occurrence of oligo-anovulation and PCOM (via ovarian volume), in the absence of hyperandrogenism. Given that the Non-HA phenotype (Rotterdam Phenotype D) relies heavily on the exclusion of other causes of anovulation, we rigorously excluded alternative diagnoses, including Functional Hypothalamic Amenorrhoea (FHA), as mandated by the study protocol. Exclusion was based on the absence of key FHA indicators—such as a history of significant energy deficit (e.g., disordered eating, excessive exercise), low or normal gonadotrophin levels (contrasting with the typical PCOS profile), and profoundly low oestradiol—and through comprehensive screening for other endocrine disorders (25, 27). This rigorous selection ensures that the Non-HA cohort represents a valid PCOS phenotype defined by neuroendocrine dysfunction, rather than misclassified FHA.

Endocrine profiling revealed that the HA phenotype is characterised by a higher gonadotrophin drive. Both basal FSH (5.35 ± 1.28 mIU/mL vs. 4.76 ± 1.28 mIU/mL; *P* < 0.001) and LH levels (8.05 ± 4.66 mIU/mL vs. 6.08 ± 3.69 mIU/mL; *P* < 0.001) were significantly elevated in HA patients compared to their Non-HA counterparts. However, the LH/FSH ratio remained similar between groups (*P* = 0.882). As anticipated by the stratification criteria, serum testosterone was markedly higher in the HA group (*P* < 0.001). No significant differences were observed in serum oestradiol (*P* = 0.314) or prolactin levels (*P* = 0.064). Notably, the HA phenotype was strongly linked to a more pronounced degree of metabolic dysfunction. As illustrated in **Figure 2E**, whilst the HOMA-IR levels in the Non-HA group (2.82 ± 1.65) also exceeded the conventional pathological threshold of 2.5, it remained significantly lower than that observed in the HA group (3.79 ± 2.25; *P* < 0.001). This quantitative gradient underscores that insulin resistance is a feature across the PCOS spectrum, yet its severity is substantially amplified within the hyperandrogenic phenotype.

### Interrelationships between metabolic indices and reproductive hormones

**Figure 3:**
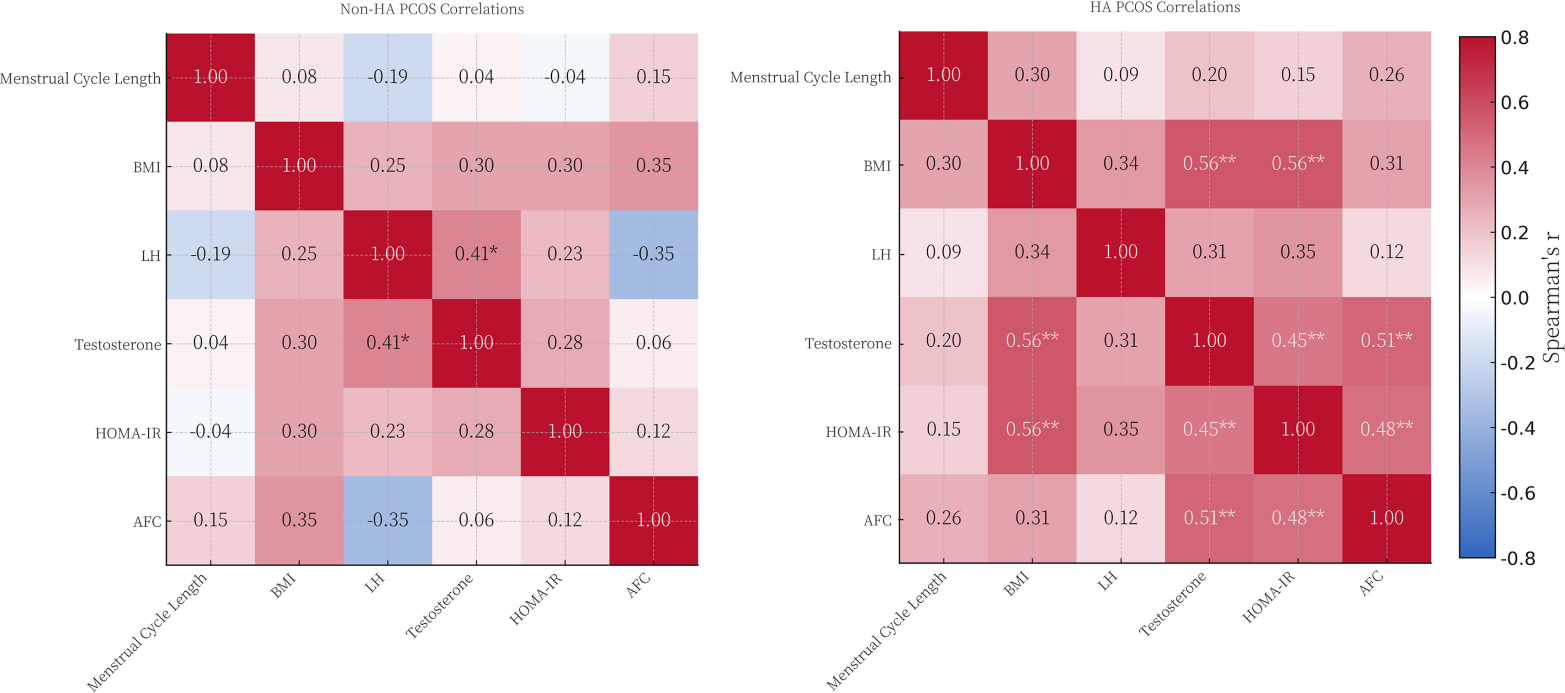
Spearman’s rank correlation matrices of endocrine and metabolic parameters stratified by PCOS phenotype. The heatmaps illustrate the pairwise relationships between Menstrual Cycle Length, BMI, LH, T, HOMA-IR, and Antral Follicle Count (AFC). (Left Panel) Correlation matrix for the Non-Hyperandrogenic (Non-HA) PCOS subgroup. (Right Panel) Correlation matrix for the Hyperandrogenic (HA) PCOS subgroup. The diverging colour gradient indicates the correlation coefficient ( r), ranging from deep blue (strong negative correlation, −0.8) to deep red (strong positive correlation, +0.8). Statistical Significance: * *P* < 0.05, ** *P* < 0.01. Unmarked cells indicate non-significant correlations.

To delineate the distinct pathophysiological architectures driving each phenotype, we performed Spearman’s rank correlation analyses among key anthropometric, hormonal, and ovarian parameters. The resulting matrices, stratified by clinical subtype, revealed fundamentally divergent regulatory patterns (**Figure 3**). In the Non-HA cohort (**Figure 3**, Left Panel), the endocrine profile appeared largely uncoupled from systemic metabolism. We observed a specific positive correlation between LH concentrations and Testosterone (*r* = 0.41, *P* < 0.05). Notably, neither BMI nor HOMA-IR showed significant associations with reproductive hormones or Antral Follicle Count (AFC) in this subgroup. This isolation of metabolic factors suggests that the mild androgenic activity in Non-HA patients is predominantly maintained by neuroendocrine mechanisms (i.e., LH-dependent steroidogenesis) rather than by insulin-mediated pathways.

Conversely, the HA phenotype exhibited a pronounced metabolic-endocrine coupling (**Figure 3**, Right Panel). Testosterone levels demonstrated robust positive correlations with both BMI (*r* = 0.56, *P* < 0.01), and HOMA-IR (*r* = 0.56, *P* < 0.01), substantiating the biological link where adiposity and insulin resistance act as potentiators of ovarian androgen synthesis. Furthermore, Testosterone was significantly positively correlated with AFC (*r* = 0.51, *P* < 0.01), linking hyperandrogenism directly to the severity of follicular arrest. These disparate correlation profiles corroborate the hypothesis that while Non-HA represents a phenotype driven by local or central gonadotropic factors, the HA phenotype is characterised by a pathogenic synergy where metabolic dysregulation exacerbates hyperandrogenism and ovarian morphology.

### Independent determinants of the hyperandrogenic phenotype and the mechanistic role of insulin resistance

To ascertain whether the observed metabolic and hormonal elevations functioned as independent drivers of the hyperandrogenic phenotype rather than mere confounders of adiposity, we conducted univariate and multivariate logistic regression analyses (**Table 2**). In the univariate analysis, both LH (*P* < 0.001) and HOMA-IR (*P* < 0.001) were identified as significant predictors of hyperandrogenism. Crucially, after adjusting for Age and BMI in the multivariate model, HOMA-IR retained its status as a robust independent predictor (adjusted Odds Ratio [aOR] = 1.35, 95% CI: 1.11 – 1.64, *P* = 0.003), comparable to the predictive power of LH (aOR = 1.09, 95% CI: 1.02 – 1.17, *P* = 0.012). Of particular clinical importance, BMI failed to demonstrate independent predictive value in the adjusted model (aOR = 0.98, *P* = 0.682). This lack of significance, juxtaposed with the robustness of HOMA-IR, suggests that the metabolic contribution to the hyperandrogenic phenotype is mediated primarily through insulin resistance per se, operating independently of body mass index.

**Table 2:** Univariate and multivariate logistic regression analysis identifying independent predictors of the HA phenotype in PCOS patients.

Variables	Univariate analysis		Multivariate analysis^a^	
	OR (95% CI)	*P*-value	aOR (95% CI)	*P*-value
Age (years)	0.95 (0.89 – 1.01)	0.072	0.96 (0.90 – 1.03)	0.215
BMI (kg/m²)	1.02 (0.95 – 1.09)	0.635	0.98 (0.90 – 1.07)	0.682
LH (mIU/ml)	1.12 (1.05 – 1.19)	**<0.001**	1.09 (1.02 – 1.17)	**0.012**
HOMA-IR	1.48 (1.22 – 1.79)	**<0.001**	1.35 (1.11 – 1.64)	**0.003**

The multivariate model was adjusted for potential confounders, including age and BMI. Notably, while LH and HOMA-IR emerged as robust independent predictors in the adjusted model, BMI did not demonstrate a statistically significant association (*P* = 0.682). This finding underscores that the contribution of HOMA-IR to the hyperandrogenic phenotype operates independently of adiposity. Data are presented as Odds Ratios (OR) and adjusted Odds Ratios (aOR) with 95% Confidence Intervals (CI). Bold values indicate statistical significance (*P* < 0.05). **^a^** Adjusted for Age and BMI.

**Figure 4:**
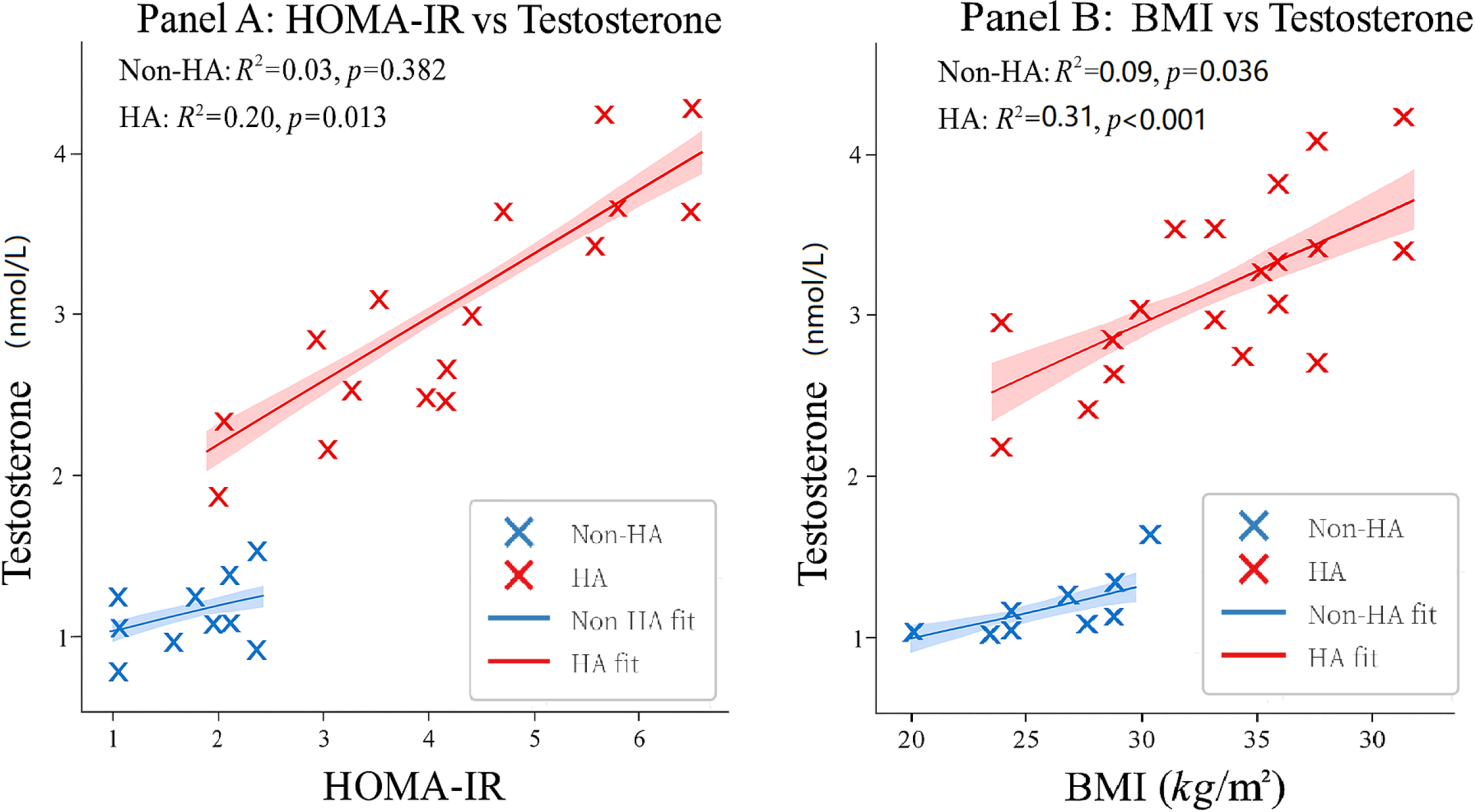
Differential impact of metabolic drivers on serum testosterone levels across PCOS phenotypes. Scatter plots with superimposed linear regression lines illustrating the relationship between total testosterone and **(A)** Homeostatic Model Assessment for HOMA-IR and **(B)** BMI. The study population is stratified into Non-HA (blue crosses) and HA (red crosses) subgroups. Solid lines represent the line of best fit, and shaded bands indicate the 95% confidence intervals (95% CI). **(A)** A significant positive linear trajectory is observed between HOMA-IR and testosterone specifically within the HA phenotype ( $R^{2}=0.20,P=0.013$), whereas no significant association exists in the Non-HA cohort ( P=0.382), highlighting the metabolic-hormonal coupling in severe PCOS. **(B)** While BMI correlates with testosterone in both subgroups, the HA phenotype exhibits a markedly stronger association ( $R^{2}=0.31,P<0.001$) compared to the Non-HA group ( $R^{2}=0.09,P=0.036$), suggesting a heightened sensitivity to adiposity-related androgen excess in the hyperandrogenic phenotype. Statistical parameters (coefficient of determination $R^{2}$ and P-value) are displayed within each panel.

To further elucidate the mechanistic coupling between these metabolic indices and androgen excess, we performed stratified linear regression analyses (**Figure 4**). **Figure 4A** reveals a striking divergence in the impact of insulin resistance: a significant positive linear trajectory exists between HOMA-IR and total testosterone specifically within the HA subgroup ( $R^{2}=0.20,P=0.013$), whereas no such association is observed in the Non-HA cohort. This steeper slope in the HA phenotype supports the hypothesis of a heightened sensitivity of ovarian theca cells to insulin stimulation in these patients. Parallel analysis of BMI (**Figure 4B**) provides a nuanced perspective. Whilst **Table 2** indicates that BMI cannot independently distinguish between phenotypes (due to overlapping distributions), **Figure 4B** demonstrates that within the HA phenotype, increasing BMI significantly exacerbates testosterone levels ( $R^{2}=0.31,P<0.001$). This contrasts with the weaker association in the Non-HA group ( $R^{2}=0.09$), indicating that while obesity is not the sole determinant of the phenotype, it acts as a potent amplifier of androgen excess specifically in women predisposed to hyperandrogenism. Collectively, these data position HOMA-IR as a critical, independent biomarker for risk stratification, offering diagnostic value beyond simple anthropometric measurements.

## Discussion

This study provides a comprehensive evaluation of clinical, hormonal, and metabolic parameters across control, Non-HA, and HA subgroups of PCOS, offering critical insights into the heterogeneous clinical presentations and underlying pathophysiological mechanisms of the disorder. International guidelines and contemporary consensus increasingly frame PCOS as a complex, multifaceted syndrome, defined by its cardinal features of ovulatory dysfunction, hyperandrogenism, and polycystic ovarian morphology, with a metabolic risk profile that is intrinsically linked to phenotypic presentation (28–30). The most recent 2023 International Evidence-based Guideline consolidates and refines this framework, re-emphasising the cautious interpretation of isolated biochemical markers (such as the LH/FSH ratio) while further delineating the broad reproductive–metabolic spectrum of the syndrome (25, 31). Our data not only align with this framework but, crucially, extend it by demonstrating that the HA and Non-HA phenotypes appear to be underpinned by distinct pathophysiological networks.

### Divergent pathophysiological drivers: neuroendocrine vs. metabolic

A pivotal finding of our study, elucidated by the stratified correlation analyses (**Figure 3**), is the marked divergence in regulatory mechanisms between phenotypes. We observed that in the Non-HA subgroup, menstrual cycle prolongation correlated significantly with LH levels but exhibited no association with metabolic indices. This suggests that the Non-HA phenotype is predominantly a neuroendocrine disorder, driven by perturbed GnRH pulsatility and preferential LH secretion, operating largely independently of insulin resistance. This aligns with established mechanistic models linking rapid GnRH pulse frequency to arrested follicular development (32).

Conversely, the HA phenotype displayed a distinct metabolic-endocrine coupling, where cycle length and testosterone levels were strongly associated with HOMA-IR. This supports the prevailing view that hyperandrogenic phenotypes carry a more adverse endocrine and metabolic profile than non-hyperandrogenic presentations. The progressive gradient of cycle prolongation observed in our cohort—longest in the HA group—is likely underpinned by the synergistic deleterious effects of LH and hyperinsulinaemia on granulosa cell function and follicular arrest (1, 4, 7–9, 20, 33, 34).

### The independent role of insulin resistance in hyperandrogenism

While insulin resistance is a known feature of PCOS, a critical question remains regarding its mechanistic independence from obesity. Our multivariate regression analysis (**Table 2**) provides a definitive answer: HOMA-IR remained a robust, independent predictor of the hyperandrogenic phenotype even after adjusting for age and BMI, whereas BMI itself lost statistical significance. This finding is clinically profound. It challenges the notion that metabolic dysfunction in PCOS is merely a consequence of adiposity. Instead, our data suggest that insulin resistance acts as a primary pathogenic driver, amplifying theca cell androgen synthesis via intrinsic pathways (35, 36). The linear regression models (**Figure 4**) further nuance this relationship: while BMI acts as an amplifier of testosterone levels specifically within the HA cohort (**Figure 4B**), the fundamental driver distinguishing the phenotype remains insulin resistance (**Figure 4A**). These findings reinforce calls for proactive metabolic risk stratification and lifestyle interventions in HA phenotypes, irrespective of BMI status (36, 37). Our data, consistent with the spectrum model of PCOS, suggest that insulin resistance is not a binary trait but exists on a continuum of severity across phenotypes (38, 39). The significantly higher HOMA-IR in HA patients (**Figure 2E**) solidifies its role as a major driver of both metabolic complications and, crucially, the reproductive dysfunction and hyperandrogenaemia that define this subgroup. Conversely, in the Non-HA phenotype, where the HOMA-IR level was lower yet still often pathological, metabolic dysfunction may play a more permissive or secondary role. Here, the clinical picture appears to be primarily characterised and initiated by neuroendocrine dysregulation, as suggested by its stronger correlation with LH rather than metabolic indices (**Figure 3**). This nuanced interpretation aligns with the complex, multifactorial aetiology of PCOS and underscores the importance of phenotype-specific management strategies.

Beyond the clinical presentation, the phenotypic divergence observed in our study likely reflects distinct underlying biological architectures. Emerging evidence suggests that the heterogeneity of PCOS is not merely symptomatic but is orchestrated by a complex interplay of genetic and epigenetic mechanisms (40). Recent large-scale genetic analyses have identified specific loci associated with PCOS that cluster into distinct “reproductive” and “metabolic” pathways. Indeed, Dapas et al. recently delineated two distinct PCOS subtypes based on genetic clustering: a “reproductive” subtype characterised by higher LH and SHBG levels, and a “metabolic” subtype characterised by higher BMI and insulin resistance (40, 41). It is plausible that our HA phenotype aligns with this metabolic driver, whereas the Non-HA phenotype may be more strongly influenced by loci governing gonadotropin secretion. Furthermore, epigenetic modifications, potentially induced by prenatal androgen exposure or *in utero* metabolic disturbances, may further entrench these phenotypes by reprogramming gene expression patterns related to steroidogenesis and insulin sensitivity, thereby determining the individual’s trajectory towards a predominant metabolic or reproductive presentation (42–44).

### Gonadotrophin dynamics and diagnostic nuances

Although elevated LH or LH/FSH ratios have historically been associated with PCOS, the diagnostic value is variable and assay-dependent. In our cohort, whilst LH levels were broadly elevated across the PCOS spectrum compared with controls, the LH/FSH ratio failed to reliably discriminate between the HA and Non-HA phenotypes. This outcome supports the international guideline’s caution against using the LH/FSH ratio as a stand-alone diagnostic criterion (31, 37). However, the evaluation of gonadotrophin dynamics remains clinically indispensable, particularly for the rigorous differential diagnosis between Non-HA and Functional Hypothalamic Amenorrhoea (FHA). This distinction presents a significant challenge, as both conditions manifest with oligo-anovulation and non-hyperandrogenic profiles. As highlighted in a recent update by Ott et al., accurately differentiating these entities requires a nuanced integration of hormonal and metabolic parameters (45). Unlike the suppressed or low-normal LH pulsatility typical of FHA (driven by central energy deficits), our Non-HA subgroup maintained robust LH levels significantly higher than controls, reflecting the distinct neuroendocrine hyperactivity inherent to PCOS. Furthermore, metabolic profiling serves as a crucial discriminator; whilst FHA is characterised by a hypo-metabolic state (e.g., profound insulin sensitivity, low leptin), the Non-HA phenotype—even in the absence of overt insulin resistance—does not exhibit the biochemical signatures of chronic energy deprivation. Thus, the sustained LH elevation observed in our Non-HA subjects serves as a vital biomarker to substantiate the PCOS diagnosis and rule out hypothalamic suppression, reinforcing the validity of our Non-HA cohort selection.

### Ovarian morphology and prolactin assessment

Regarding ovarian morphology, our results reveal that HA patients exhibited a more severe “polycystic” phenotype (higher prevalence of bilateral PCOS) compared to the Non-HA group. This aligns with the biological model where local androgen excess, potentiated by insulin, promotes early follicular recruitment but arrests selection, leading to the characteristic accumulation of antral follicles (46). Regarding prolactin, our observation of mild elevations in the PCOS group compared to controls warrants a nuanced interpretation. Whilst differential diagnosis must exclude organic hyperprolactinaemia, mild elevations are frequently artefactual or stress-related (38). Our real-world data support contemporary practice to confirm true monomeric prolactin through methods such as post-rest sampling and polyethylene glycol (PEG) precipitation before considering pituitary imaging, particularly in populations with otherwise typical PCOS features (47).

### Methodological considerations and limitations

The bidirectional relationship between insulin and androgen levels adds a layer of complexity (48–51). While HOMA-IR provided a practical measure of insulin resistance in this large-scale study, we acknowledge its limitations compared to the gold-standard hyperinsulinaemic-euglycaemic clamp. However, prior studies have demonstrated that HOMA-IR correlates well with clamp-derived measures in PCOS populations, supporting its validity in epidemiological contexts (13, 14). Furthermore, in a clinical outpatient setting, the clamp technique is impractical, and the Oral Glucose Tolerance Test (OGTT) poses compliance challenges; thus, HOMA-IR represents a balanced choice between accuracy and feasibility. A notable strength of our study is the parallel, within-subgroup control comparisons and the rigorous statistical stratification (**Table 2**), which allowed us to disentangle the effects of adiposity from intrinsic metabolic dysfunction. Limitations include the cross-sectional design which precludes causal inference, and the use of the 2003 Rotterdam threshold for follicle number per ovary (FNPO ≥ 12) rather than the stricter thresholds proposed in current guidelines (FNPO ≥ 20), reflecting the clinical standards at the time of recruitment (37). Finally, we acknowledge certain constraints in our metabolic and hormonal phenotyping. The retrospective nature of our study precluded the assessment of waist-to-hip ratio (WHR) and sex hormone-binding globulin (SHBG) levels. The absence of WHR limits our ability to fully capture visceral adiposity, which is a more sensitive marker of metabolic dysfunction than BMI alone. Similarly, without SHBG, we could not calculate the Free Androgen Index (FAI), potentially masking the true extent of bioavailable androgenicity. Consequently, the metabolic and androgenic burden in our cohort may have been underestimated, suggesting that the spectrum of metabolic risk we observed could be even more pronounced in a prospective setting.

## Conclusion

By situating our findings within the contemporary evidence base, we confirm that hyperandrogenism delineates a more dysmetabolic and dysgonadotrophic phenotype in PCOS. Crucially, we identify that this phen assay-dependantotype is driven by insulin resistance independent of obesity, and that the underlying regulatory networks differ significantly between HA (metabolic-driven) and Non-HA (neuroendocrine-driven) subgroups. These insights advocate for a phenotype-specific approach to management, necessitating a paradigm shift that prioritises insulin-sensitising strategies in hyperandrogenic patients and refining care pathways in reproductive endocrinology.

## Data Availability

The raw data supporting the conclusions of this article will be made available by the authors, without undue reservation.
