# Supplementary material for: Divergent pathophysiological drivers of polycystic ovary syndrome: insulin resistance independently fuels the hyperandrogenic phenotype whilst neuroendocrine factors dominate non-hyperandrogenic presentations
Source: Front Endocrinol (Lausanne). 2026 Feb 4;17:1758861. doi: 10.3389/fendo.2026.1758861 (PMC12913134; doi:10.3389/fendo.2026.1758861)
Supplement: Supplementary file 1 [file Table1.docx]

**Supplement table 1: Comparative analysis of clinical and biochemical characteristics stratified by hyperandrogenism status (HA vs. Non-HA).** P-values represent comparisons between Control and PCOS groups within the specific sub-cohort (Non-HA or HA). The sample size of the HA Control group is limited (n=13); results should be interpreted with appropriate caution. **Bold** values indicate statistical significance (P < 0.05).

|  | Non-HA Cohort | |  | HA Cohort | |  |
| --- | --- | --- | --- | --- | --- | --- |
|  | Control group (n=131) | PCOS group (n=81) | P | Control group (n=13) | PCOS group (n=220) | P |
| Age | 30.61±4.96 | 29.1±3.84 | 0.123 | 29.62±3.33 | 28.83±4.05 | 0.492 |
| Menstrual cycles (days) | 30.57±2.06 | 62.05±21.28 | **<0.001** | 29.23±2.35 | 63.12±20.26 | **<0.001** |
| BMI(kg/m^2^) | 20.80±2.81 | 22.54±3.67 | **0.038** | 22.19±3.97 | 22.64±3.25 | 0.637 |
| FSH(mIU/ml) | 5.04±1.28 | 4.68±1.29 | 0.181 | 5.48±1.57 | 5.37±1.25 | 0.767 |
| LH(mIU/ml) | 4.17±1.69 | 6.88±3.85 | **<0.001** | 5.21±1.86 | 8.4±4.82 | **0.005** |
| LH/FSH | 0.87±0.38 | 1.75±2.30 | **0.035** | 1.00±0.40 | 1.59±0.87 | **0.004** |
| E2(pmmol/L) | 130.12±54.01 | 178.26±336.28 | 0.423 | 141.32±54.28 | 135.95±46.47 | 0.634 |
| PRL(mIU/L) | 367.84±147.83 | 387.55±220.14 | 0.642 | 370.45±102.04 | 455.3±318.52 | 0.250 |
| T(nmol/L) | 0.92±0.39 | 1.27±0.39 | **<0.001** | 2.52±0.47 | 3.24±1.18 | **0.009** |
| HOMA_IR | 1.56±0.44 | 3.27±1.71 | **<0.001** | 1.81±0.47 | 3.92±2.28 | **<0.001** |

**Supplementary Table 2. Comparative analysis of clinical, hormonal, and metabolic characteristics between Hyperandrogenic (HA) and Non-Hyperandrogenic (Non-HA) PCOS phenotypes.** Data are expressed as mean ± standard deviation (SD). Ovarian morphology (categorical data) was compared using the Chi-square test. **Bold** text denotes statistical significance (P < 0.05).

|  | Non-HA group (n=49) | HA group (n=252) | t | *P* |
| --- | --- | --- | --- | --- |
| Age | 29.7±4.2 | 28.8±4.0 | 1.822 | 0.069 |
| Menstrual cycles (days) | 54.2±22.3 | 61.2±21.5 | -2.661 | **0.008** |
| Hormone Indicators | | | | |
| FSH(mIU/ml) | 4.76±1.28 | 5.35±1.28 | -3.884 | **<0.0001** |
| LH(mIU/ml) | 6.08±3.69 | 8.05±4.66 | -4.174 | **<0.0001** |
| LH/FSH | 1.51±2.11 | 1.53±0.85 | -0.148 | 0.882 |
| E2(pmol/l) | 167.59±304.18 | 136.66±46.8 | 1.012 | 0.314 |
| PRL(mIU/l) | 383.41±209.23 | 444.5±301.62 | -1.855 | 0.064 |
| T(nmol/l) | 1.16±0.42 | 3.08±1.21 | -22.175 | **<0.0001** |
| HOMA_IR | 2.82±1.65 | 3.79±2.25 | -4.319 | **<0.0001** |
|  |  |  |  |  |
| Polycystic Ovary Count (Score) | N(%) |  | χ^2^ | *P* |
| 0 | 15（29.7） | 32（13.0） | 17.389 | **<0.0001** |
| 1 | 10（20.9） | 36（14.6） |  |  |
| 2 | 24（49.5） | 178（72.4） |  |  |

**Note:** The HA phenotype in this analysis includes patients with biochemical hyperandrogenism and/or clinical hyperandrogenism (hirsutism/acne). Thus, the sample size ($n=252$) is larger than the group defined by serum testosterone alone ($n=220$). Polycystic Ovary Count (Score) is defined based on the morphological dominance: 0 = Volume-dominant phenotype (Ovarian Volume ≥ 10 mL with FNPO < 12); 1 = Follicle-dominant phenotype (FNPO ≥ 12 with Ovarian Volume < 10 mL); 2 = Mixed phenotype (meeting both criteria). Bold text denotes statistical significance (P < 0.05).
